# Current and Future of “Turn‐On” Based Small‐Molecule Copper Probes for Cuproptosis

**DOI:** 10.1002/open.202300078

**Published:** 2023-09-13

**Authors:** Ting‐En Peng, Feng Qiu, Yunwei Qu, Changmin Yu, Xiamin Cheng, Lin Li

**Affiliations:** ^1^ Key Laboratory of Flexible Electronics (KLOFE) & Institute of Advanced Materials (IAM) Jiangsu National Synergetic Innovation Center for Advanced Materials (SICAM) Nanjing Tech University Nanjing 211816 China; ^2^ Institute of Advanced Synthesis School of Chemistry and Molecular Engineering Jiangsu National Synergetic Innovation Center for Advanced Materials (SICAM) Nanjing Tech University Nanjing 211816 China; ^3^ The Institute of Flexible Electronics (IFE, Future Technologies) Xiamen University Xiamen 361005 China

**Keywords:** bioimaging, copper, cuproptosis, fluorescent probe, fluorescence turn on

## Abstract

Increasing evidence shows that abnormal copper (Cu) metabolism is highly related to many diseases, such as Alzheimer's disease, Wilson's disease, hematological malignancies and Menkes disease. Very recently, cuproptosis, a Cu‐dependent, programmed cell death was firstly described by Tsvetkov et al. in 2022. Their findings may provide a new perspective for the treatment of related diseases. However, the concrete mechanisms of these diseases, especially cuproptosis, remain completely unclear, the reason of which may be a lack of reliable tools to conduct highly selective, sensitive and high‐resolution imaging of Cu in complex life systems. So far, numerous small‐molecular fluorescent probes have been designed and utilized to explore the Cu signal pathway. Among them, fluorescence turn‐on probes greatly enhance the resolution and accuracy of imaging and may be a promising tool for research of investigation into cuproptosis. This review summarizes the probes developed in the past decade which have the potential to study cuproptosis, focusing on the design strategies, luminescence mechanism and biological‐imaging applications. Besides, we put forward some ideas concerning the design of next‐generation probes for cuproptosis, aiming to tackle the main problems in this new field. Furthermore, the prospect of cuproptosis in the treatment of corresponding diseases is also highlighted.

## Graphical abstract

Cuproptosis, firstly proposed in 2022, is considered a newly identified type of programmed cell death caused by excessive copper, which provides a new perspective for the treatment of related diseases. Unfortunately, its specific mechanism remains unclear. This article summarizes available probes with a potential for studying cuproptosis that have been disclosed over the past decade.

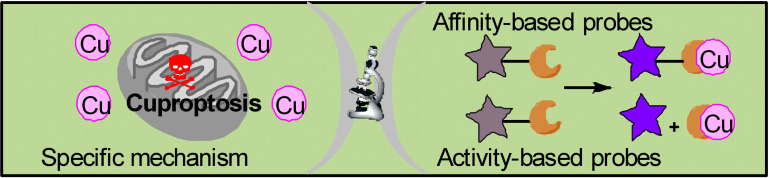

## 1 Introduction

### 1.1 Functions and metabolism of copper (Cu)

Metals play a vital role in the life of organisms as they are usually involved in essential physiological processes. However, abnormal metabolism of metal elements often induces dysfunction and diseases. For instance, iron involves in oxygen transport^[1]^ and even causes cell death, ferroptosis.^[2]^ As the third‐most abundant transition metal in the body, copper (Cu), serves as an essential cofactor for enzymes which mediate a range of essential cellular functions,^[3]^ such as neurotransmitter synthesis, respiration and free radical scavenging.[^4^, ^5^] Besides the role of static cofactor, it also acts as a dynamic signal species in protein kinases, lipolysis and regulation of potassium channels.^[6]^ Abnormal Cu metabolism has caused neurodegenerative diseases, Wilson's disease,^[7]^ Menkes disease^[8]^ and so on. Thus, it's highly desirable to detect Cu.

Cu has two common oxidation states, Cu(I) and Cu(II). In the digestive process, Cu(II) from food is reduced to Cu(I) by reductase, then absorbed by the intestine via copper transporter 1 (CTR1), transported to various systems throughout the body through the portal vein circulation and mainly stored in the liver. Serum albumin (SA) as well as α_2_‐Macroglobulin (α_2_M), Cu carrier in blood, transported Cu(II) to cells.^[9]^ When entering the cell, Cu is strictly regulated by metal transporters and chaperones in the cell to prevent the existence of free Cu in toxic form.^[10]^ Because Cu is rapidly precipitated or oxidized to cuprous oxide by intramolecular reactive oxygen species in the cytoplasm, and it often coordinates with low molecular weight biomolecules or proteins to form complexes, the concentration of free Cu in the cytoplasm is as low as 10^−15^–10^−20^ M under normal physiological conditions,^[11]^ which contributes to the Cu homeostasis and the buffering of Cu between cells and the external environment. Cu is delivered to various subcellular organelles by chaperones.^[12]^ For example, it is transported by cytochrome oxidase 17 to mitochondrion.^[13]^ Excess cellular Cu could be exported by Cu‐ATPases ATP7 A/B.^[14]^

According to the affinity of coordination, Cu exists in static pool or labile pool.[^15^, ^16^] The former tightly binds to proteins. But the latter loosely binds to ligands, which plays a vital role in the exchange as well as buffering of Cu between cells and the external environment. Cu(I) plays a dominant role in the labile copper pool and cytoplasm. Labile copper can lead to oxidative stress and form reactive oxygen species under pathological conditions through Fenton‐type reactions.^[17]^ Therefore, the labile copper species are main targets in the detection.

### 1.2 Cuproptosis

**Figure 1:**
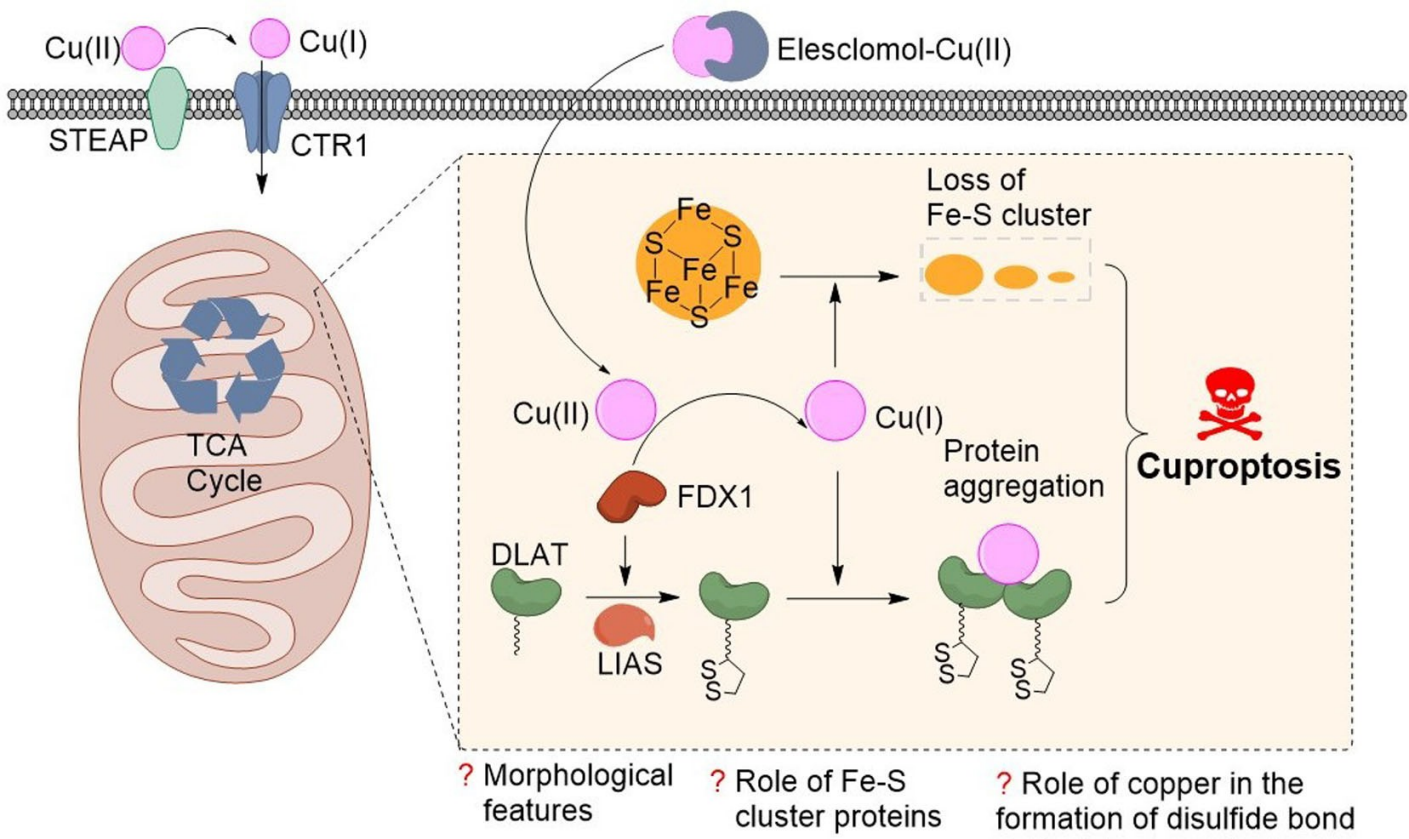
Schematic diagram of Cu‐induced cell death.

Very recently, cuproptosis firstly raised by Tsvetkov et al.^[18]^ in 2022 remains a new programmed cell death, which induces by excess Cu and differs in apoptosis and ferroptosis (Figure 1). The process of cuproptosis is cellularly regulated. Firstly, the Cu ionophore elesclomol transports Cu(II) to the reductase encoded by ferredoxin 1 (FDX1) that then reduces it to the more toxic Cu(I). Secondly, FDX1 is a upstream regulator of protein lipoylation. It cooperates with lipoyl synthase (LIAS) to enable dihydrolipoamide *S*‐acetyltransferase (DLAT) to be equipped with a disulfide bond, which triggers DLAT oligomerization. Finally, Cu directly binds to the lipoylated components of the tricarboxylic acid (TCA) cycle during mitochondrial respiration, leading to the aggregation of lipid acylated proteins and downregulation of iron‐sulfur cluster protein expression, thereby inducing proteotoxic stress and ultimately cell death. Importantly, Tsvetkov and co‐workers demonstrated that the mechanism of cuproptosis also applied in vivo.

Unfortunately, some questions concerning the specific mechanism of cuproptosis remain unknown. In addition, the morphological features of cuproptosis remain unclear. Last but not least, whether reduced Fe−S cluster proteins promote Cu‐mediated cell death and the relationship between Cu and disulfide bond formation mechanisms serve as uncharted.^[19]^

To address these questions, there is an urgent need for convenient and sensitive tools for analyzing possible biomarkers of cuproptosis, such as metal ions, enzymes, and small biomolecules. Particularly, the biodistribution, oxidative state, coordination of Cu is the primary issue of concern. There are many new techniques emerging for Cu analysis such as inductively coupled plasma mass spectrometry (ICP‐MS),^[20]^ atomic absorption spectroscopy (AAS),^[21]^ and X‐ray fluorescence microscopy (XFM).^[22]^ However, these techniques are difficult to non‐invasively measure the analyst in live cells. In view of the recent achievements of fluorescent probes for visualizing Cu in living organisms,^[23]^ it is strongly believed to assist to further reveal underlying mechanism of cuproptosis and open a window for the diagnosis and treatment of Cu‐related diseases.

For this purpose, there are some relative requirements that need to be addressed for developing fluorescent probes. Especially, the high correlation between Cu‐mediated cell death, protein lipoylation and FDX1 expression, turned to be lost at high concentration of elesclomol (>40 nmol). Ideally, the probe should possess the following characteristics: (1) good biocompatibility and photobleaching resistance are conducive to long‐term cell imaging, because the cuproptosis caused by excessive Cu remains programmed cell death and occurs after 24 h; (2) a good balance between lipophilicity and hydrophilicity ensures that the probe can cross the lipophilic membrane and enter the cell while maintain good distribution in aqueous medium to avoid the fluorescence quenching caused by self‐aggregation; (3) among metal ions with high biological abundance, it has a specific response to Cu; (4) strong Cu‐binding affinity, the key factor of affinity‐based probes for dynamic exchange with endogenous Cu ligands and further reporting Cu dynamics; for activity‐based probes, strong affinity for Cu‐binding would affect the relative bond which became easier to be cleaved; (5) in view of the close relation to mitochondrial respiration, high sensitivity and organelle partition detection, especially mitochondria; (6) high signal‐to‐noise ratio.

## 2 Fluorescence Turn‐on Probes for Cu

Most of fluorescence‐quenched probes for Cu detection often collect the wrong signal, which can be caused by numerous factors. In contrast, fluorescence‐enhanced probes of Cu can greatly minimize background interference originating from endogenous cellular analytes,^[24]^ which contributes to obviously improve the spatial resolution and accuracy. In the context, we have focused on the progress in fluorescence‐enhanced probes in the last decade, to guide the development of Cu probes specifically for investigating cuproptosis and other Cu‐related diseases.

**Figure 2:**
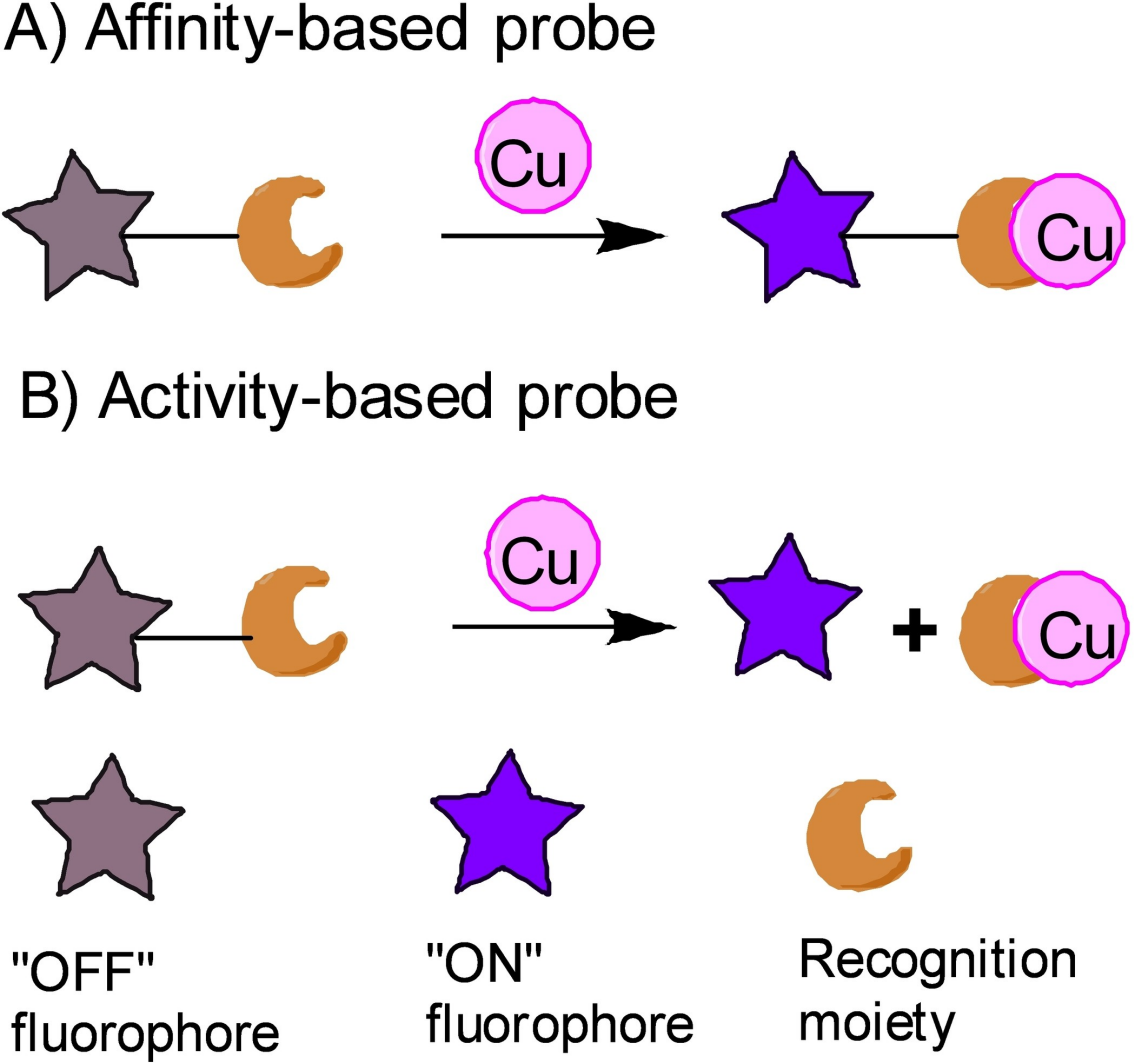
The typical structures and mechanisms of affinity‐based probe (A) and activity‐based probe (B).

Typically, a Cu‐responsive probe should include three parts: fluorophore, Cu‐responsive recognition group/receptor and linker. According to the interaction mode between probes and Cu, they can be divided into affinity‐ and activity‐based types (Figures 2A & 2B). The former refers to its fluorescence change due to reversible coordination between Cu and its receptor, while the latter refers to fluorescence change of probe due to a probe fragmentation triggered by Cu. The recognition groups for affinity‐based probes should respond specifically to Cu in the pool of bio‐related metal ions, such as azatetrathiacrown, thioether and peptide.[^25^, ^26^] To compete with endogenous Cu ligands (e. g., glutathione, protein), the receptor should also have a stronger binding affinity.^[27]^ The recognition groups for activity‐based probes should respond specifically to Cu and assist to cleave the covalent linker or caged group. They also are classified by luminescence mechanism, such as electronic transfer (ET), intramolecular charge transfer (ICT), photoinduced electron transfer (PET), and fluorescence resonance energy transfer (FRET).

### 2.1 Copper(I)

Cu cycles between intracellularly dominating Cu(I) and Cu(II) under physiological conditions. It's CTR1/2 and ATP7A/7B known as the membrane transporter that regulate Cu to import and export from the cytosol in mammalian cells. In the presence of reducing agents such as ascorbic acid, NADPH and glutathione, it is present as Cu(I) after being conveyed into the cell.^[27]^ Therefore, Cu(I) is the primary target in the cells.

#### 2.1.1 Affinity‐based probes for Cu(I)

**Figure 3:**
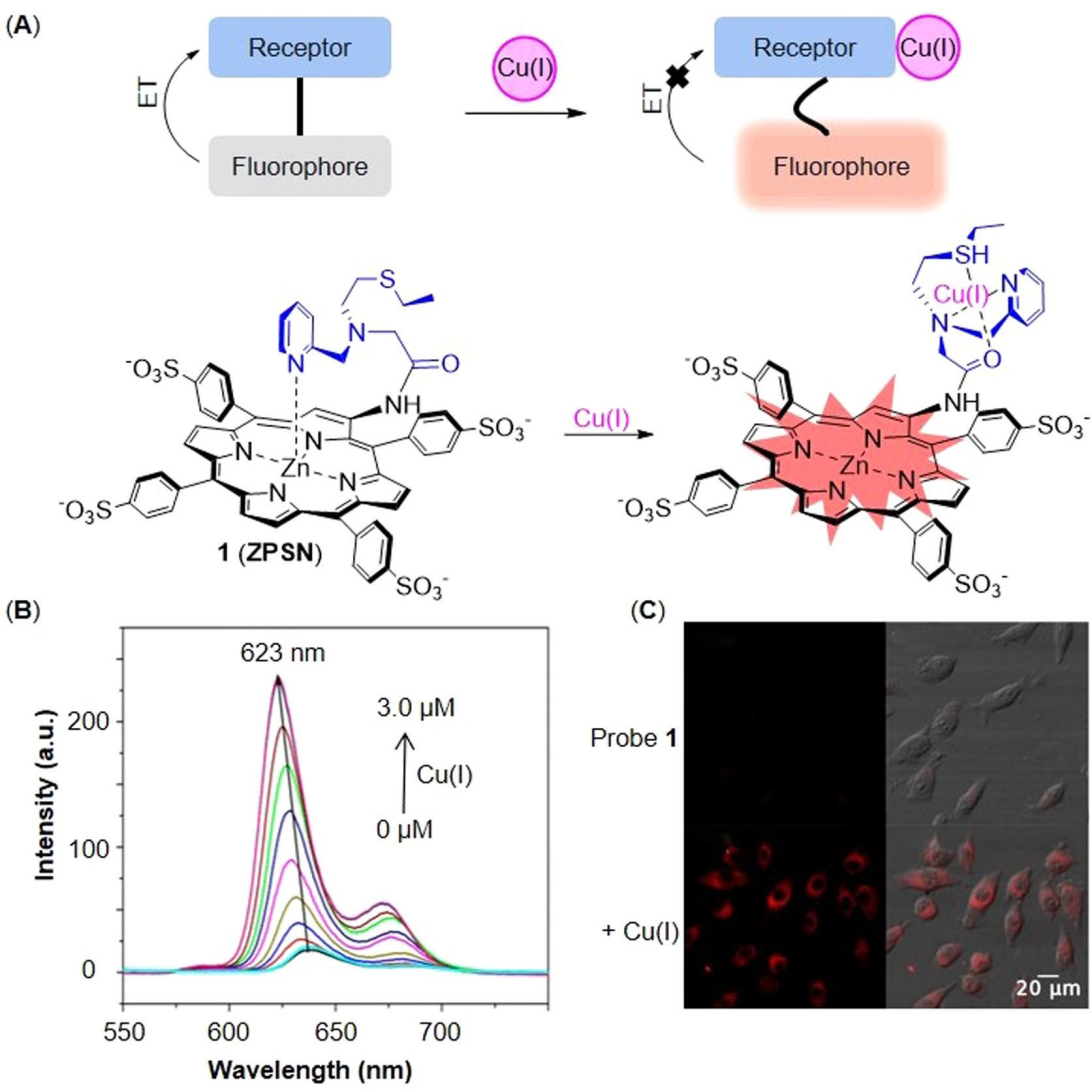
The representing of ET probes for Cu(I). (A) The structure of probe **1**, and its responsive mechanism for Cu(I); (B) The fluorescent response to Cu(I); (C) Fluorescent imaging of HeLa cells incubated with probe **1** in presence or absence of Cu(I). Reproduced with permission from Ref. [28] Copyright 2019, American Chemical Society.

ET occurs from the fluorophore to the receptor and then results in fluorescence quenching. The recognition of Cu(I) breaks the probes’ plane and inhibits ET, thus emitting fluorescence (Figure 3A). In 2019, Yi and coworkers^[28]^ designed probe **1** (named **ZPSN** in original reference) for visualizing mitochondrial Cu(I), which utilized Zn(II)‐porphyrin as the fluorophore and pyridyl‐functionalized thioether as the recognition group for Cu(I) (Figure 3A). The probe had a low detection limit at 8.2 nM (Table 1). In absence of Cu(I), the nitrogen atom in the pyridyl group coordinated with the vertical zinc nucleus, ensuring that the electrons were transferred from the excited fluorophore to the pyridine receptor, leading to fluorescence quenching. Once probe **1** bound with the Cu(I), the interaction between pyridyl‐thioether and Cu(I) restored the planarity of the porphyrin ring and intramolecular electron transfer was inhibited, and therefore the fluorescence was enhanced. Besides, the spectrum showed that the emission intensity enhanced approximately 11.8‐fold and the emission peak shifted to 623 nm with the elevated Cu(I) level (Figure 3B). Yet, the fluorescence intensity decreased whlie transition metal ion chelator, tetrakis (2‐pyridylmethyl) ethylenediamine (TPEN) was added, which proved that probe **1** had the ability of reversibly detection. Probe **1** has shown good photostability, water solubility and low cytotoxicity in the imaging of mitochondrial Cu(I) of HeLa cells (Figure 3C), which indicated that probe **1** might be used to study the specific mechanism of cuproptosis.

**Table 1:** Representative Cu(I) probes.

S. no	Structure	Luminescence mechanism	λ_ex_/λ_em_ [nm]	Response time	Limit of detection	Cell lines	Ref.
1	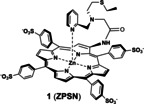	Affinity‐based: ET	430/638	5 min	8.2 nM	HeLa cells	[28]
2	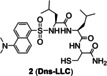	Affinity‐based: ICT	400/510	200 s (in a few minutes)	8 nM	RKO cells	[25]
3	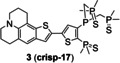	Affinity‐based: ICT	460/560	/	/	Menkes disease fibroblasts	[34]
4	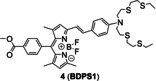	Affinity‐based: ICT	543/693	/	/	MCF‐7 cells	[26]
5	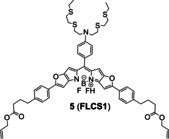	Affinity‐based: PET	610/660	/	/	SH‐SY5Y cells	[38]
6	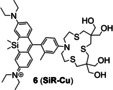	Affinity‐based: PET	620/680	/	1.2 nM	HepG2 cells	[40]
7	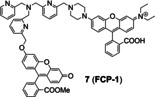	Activity‐based: FRET	458/576	30 min	16.7 nM	HEK 293T cells	[42]
8	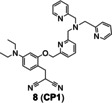	Activity‐based: /	405/480	90 min	10.8 nM	HepG2 cells	[43]
9	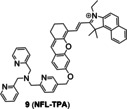	Activity‐based: FRET	650/732	/	14.4 nM	L02 cells	[44]

**Figure 4:**
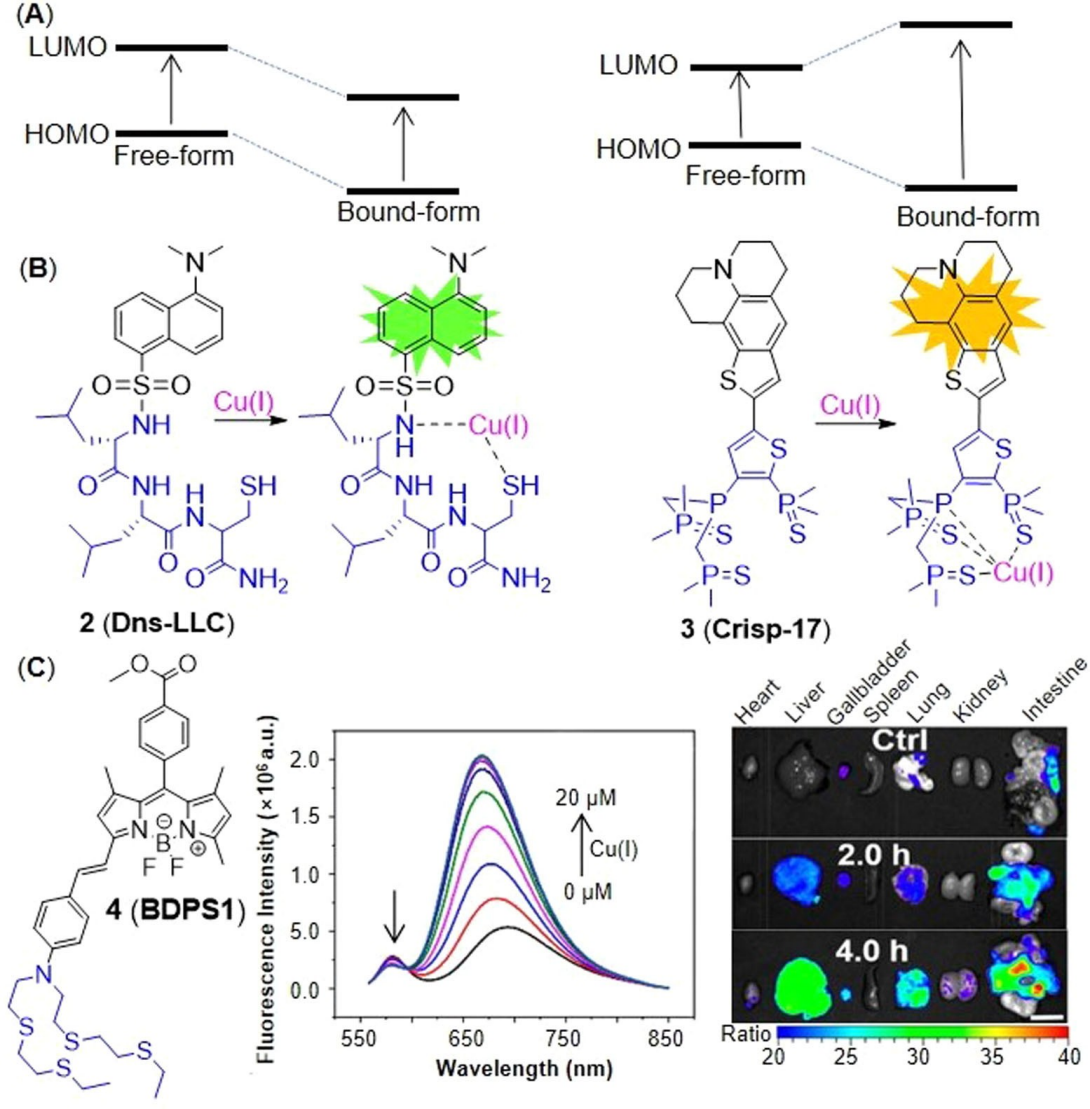
The representatives of ICT probes for Cu(I). (A) Luminescence mechanism of ICT. (B) The responsive mechanisms of probe **2** and **3** against Cu(I), respectively. (C) The structure of probe **4**, fluorescence spectra of probe/Cu(I) and in vitro imaging of mouse organs after injection of CUTX‐101 (the therapeutic agent of Menkes diseases). Reproduced with permission from Ref. [26] Copyright 2021, American Chemical Society.

The ICT probes are usually based on a large conjugated system which connects electron acceptors (A) with electron donors (D) by π linkers.^[29]^ The combination of probes and Cu(I) changes the energy levels of the highest occupied molecular orbital (HOMO) and lowest unoccupied molecular orbital (LUMO) of the conjugated system, which further alters the degree of ICT, and then triggers fluorescence. ICT can be usually divided into two situations. On one hand, when the probe combines with Cu(I), the HOMO and LUMO energy levels decrease and ICT increases, resulting the red shift of fluorescence emission peak. On the other hand, the range of HOMO and LUMO energy levels increases and then ICT decreases, leading to the blue shift of fluorescence emission peak (Figure 4A).^[30]^ In 2016, Jung's group^[25]^ developed the first probe **2** (**Dns‐LLC**) by conjugating the fluorophore danysl with *N*‐terminal peptide (Figure 4B). Peptide residues are potential Cu(I) receptors, which can endow probes with good biocompatibility, cell‐membrane permeability, good solubility and strong metal‐ion‐binding affinity. When Cu(I) was added, the amide, sulfonamide and thiol groups of receptors formed a stable complex, resulting in a 12‐fold fluorescence enhancement, which showed a blue shift of the maximum emission band at 510 nm. The recovery to Cu(I)‐free spectrum with approximately 20 equiv. dithiothreitol (DTT, the chelating agent of Cu(I)) demonstrated the strong binding affinity and reversibility of probe **2**. Probe **2** showed a dissociation constant of 12 fM, indicating a stronger binding affinity for Cu(I) than the probes with thioether‐based receptors reported previously,[^31^, ^32^, ^33^] which proved that peptide residues remained the ideal Cu(I) receptors concerning the design of probes for cuproptosis. The detection limit of 8 nM (Table 1) and high selectivity were dependent on one cysteine residue of the tripeptide receptor whose sulfhydryl group played a significant role in strongly binding to Cu(I). Additionally, due to the conjugation of a Golgi‐targeting dileucine motif, it could image the Cu(I) in the Golgi apparatus of living cells, a key organelle of Cu homeostasis. In a ratiometric fluorescent probe **3** (**crisp‐17**, Figure 4B), the fluorophore was incorporated with another Cu(I) receptor (phosphine sulfide‐stabilized phosphine, PSP), which was used in two‐photon fluorescent microscopy for visualizing attomolar (aM) Cu in Menkes and normal mutant cells.^[34]^ It not only had a binding affinity of four to six orders of magnitude higher than that of other Cu(I) probes reported previously,[^10^, ^11^] but also solved the problem that those probes could not monitor Cu(I) in presence of glutathione at normal levels. Phosphine sulfonate not only chelated Cu(I), but also stabilized the phosphine in the center which contributed the high stability of probe in air at 37 °C for 15 days. After adding Cu(I), the ICT process increased, and the peak of emission spectrum showed a redshift to 620 nm from 555 nm. The ratio‐sensing ability of probe **3** revealed that the intracellular Cu(I) was buffered to about 1 aM, which was lower than the threshold of glutathione chelating Cu(I), and further proved that glutathione did not directly participate in Cu buffering under normal physiological conditions, namely glutathione played an indirect role in Cu homeostasis. It showed that probe **3** could be used to monitor the transient change of Cu(I) and potentially detect the Cu disorders and cuproptosis.

Ratiometric probes not only reduce interferences from microenvironmental dynamic bias, photobleaching and probe concentration,^[35]^ but also enable semi‐quantification and quantification of targeting analytes.^[36]^ Xu and co‐workers^[26]^ achieved the first ratiometric near‐infrared (NIR) fluorescent probe **4** (**BDPS1**) for tracking labile Cu(I) fluctuations in mice (Figure 4C). Probe **4** consists of a boron dipyrromethene (BODIPY) fluorophore and 4‐aminostyrene whose 4‐amino group was modified with thioether as a Cu(I) receptor. Probe **4** had two emission bands, 581 nm and 693 nm. Titration of Cu(I) resulted in a blue shift of the ICT emission band to 670 nm from 693 nm. In the animal experiments, probe **4** detected a significant elevation in the labile copper pool of the upper abdomen while mice were injected intravenously and caudally with the treatment agent CUTX‐101 of Menkes diseases. In vitro proportional imaging of resected viscera showed that the labile Cu levels of spleen, heart and kidney had little change over time, while the counterpart of gallbladder, liver, lung and intestine increased in turn, which was consistent with the metabolism pathway of Cu.

**Figure 5:**
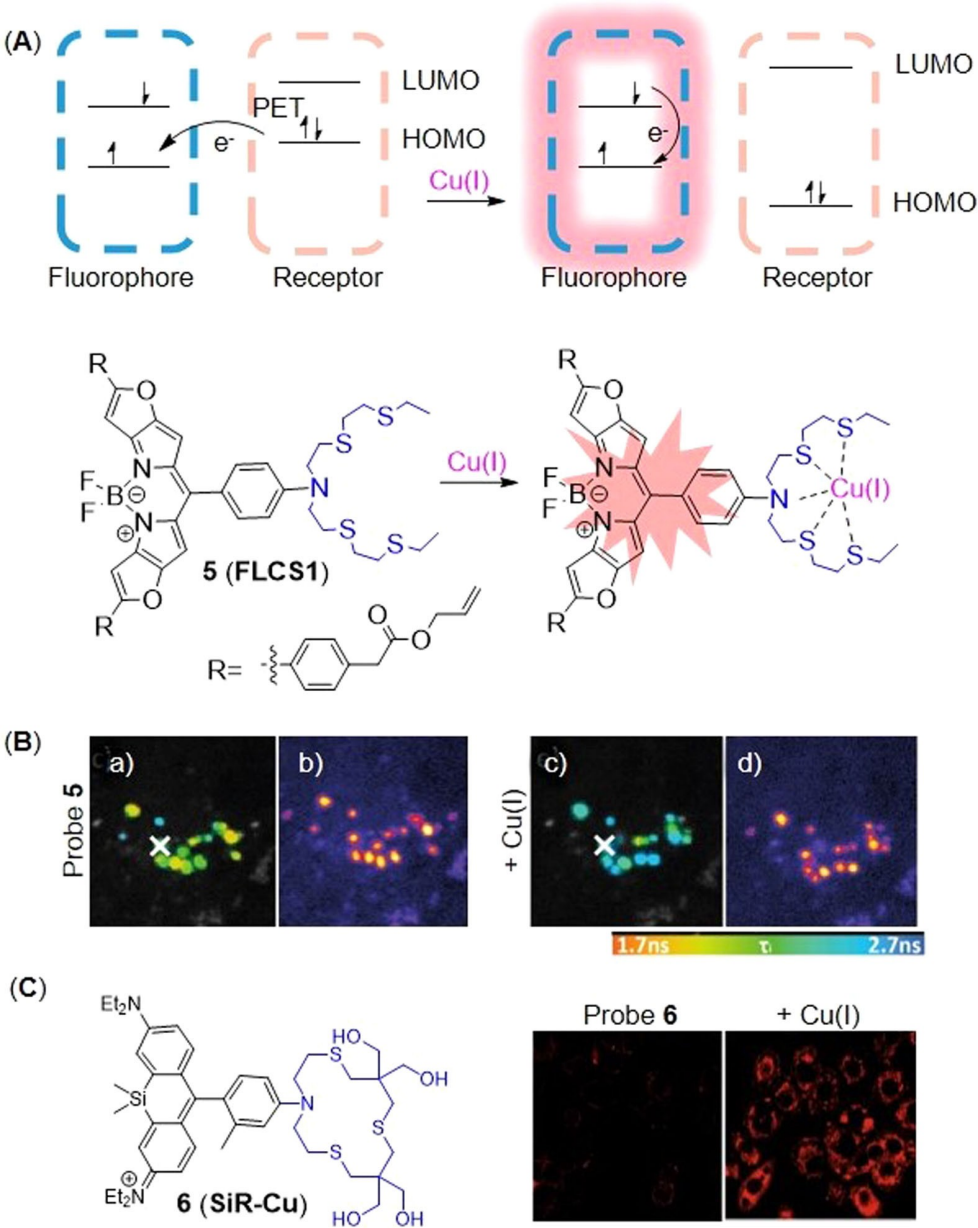
The representatives of PET probes for Cu(I). (A) The structure of probe **5**, and the responsive mechanism for Cu(I). (B) Imaging of SH‐SY5Y cells treated with probe **5**. FLIM data recorded before (a, b) and after (c, d) incubation with Cu(I), (b–d) showed fluorescence intensity images. Reproduced with permission from Ref. [38] Copyright 2021, Wiley‐VCH. (C) The structure of probe **6**, and fluorescent images of living HepG2 cells. Reproduced with permission from Ref. [40] Copyright 2021, Elsevier B.V.

The fluorescence is quenched by PET between the fluorophore and the receptor. Specifically, the electrons in the HOMO orbit of receptor transfer to HOMO orbit in the excited state of fluorophores, blocking the excited state electrons of fluorophores in LUMO orbit to return to their ground state, and therefore inhibiting fluorescence emission.^[37]^ However, once Cu(I) coordinates with receptor, it alters the orbital energy level of receptor and blocks the PET process, thus, the electrons of fluorophore can return to the ground state and simultaneously emits fluorescence (Figure 5A). In 2021, Priessner's group^[38]^ developed probe **5** (**FLCS1**) by conjugating an azatetrathia receptor with a BODIPY fluorophore, which could firstly monitor minuscule changes of Cu(I) abundance in cells by fluorescence lifetime imaging microscopy (FLIM). When PSP‐2 was added to the probe 5‐Cu(I) complex, the fluorescence intensity decreased and the addition of excessive Cu(I) to the solution would restore the fluorescence intensity, which showed the reversible process of detection. The fluorescence lifetime extended obviously from 0.39 to 3.08 ns when Cu(I) was added. Although the fluorescence intensity did not change significantly before and after treating with Cu‐GTSM, the FLIM image showed that the fluorescence lifetime of the probe **5** changed obviously (Figure 5B). It proved that FLIM was a better technique to visualize Cu species than conventional intensity‐based imaging.

NIR fluorescent probes have become powerful tools for the visualization of the spatial and temporal changes of Cu in biological systems. They not only reduce absorption and scattering of biological tissues as well as self‐fluorescence, but also possess deeper penetration and higher resolution.^[39]^ Compared with traditional rhodamine dyes, silicon substituted rhodamine dyes not only have similar stability, but also possess higher fluorescence quantum yields and long‐wavelength emission in the NIR‐spectral region. In 2021, Chai's group^[40]^ developed a hydrophilic NIR fluorescent probe **6** (**SiR‐Cu**, Figure 5C) by conjugating Si‐substituted rhodamine as a fluorophore and tetrathia‐azacrown as receptor. The probe showed a 23‐fold increase in maximum fluorescence intensity at 680 nm (Table 1) when the ratio of Cu(I) to probe was 1 : 1, as well as good photostability and water solubility. Thus silicon substituted rhodamine served as the ideal dyes for probes that were utilized to study cuproptosis. In bioimaging experiments of living HepG2 cells, it showed high selectivity as well as sensitivity to Cu(I) at physiological pH.

#### 2.1.2 Activity‐based probes for Cu(I)

**Figure 6:**
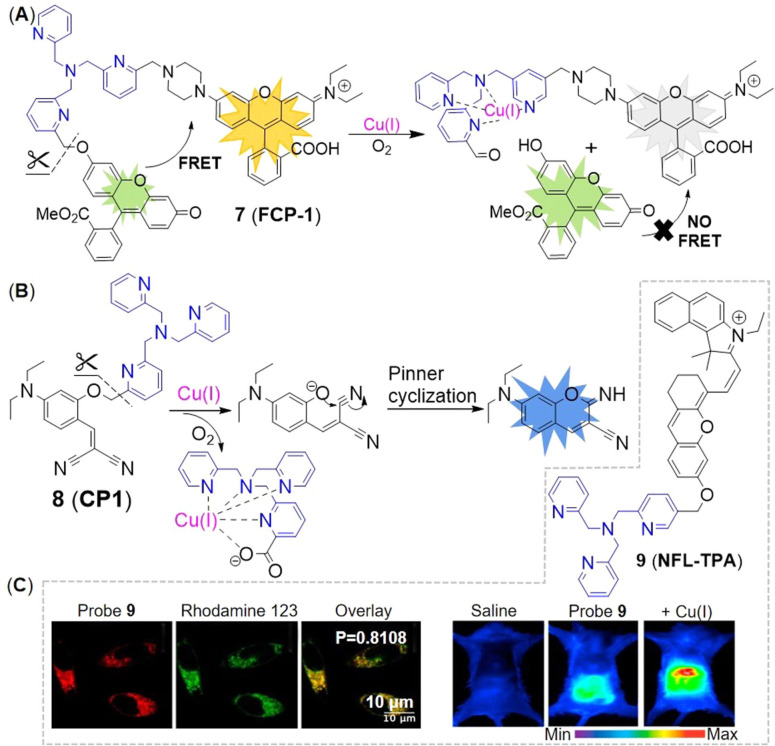
The representatives of activity‐based probes for Cu(I). (A) The structure of probe **7**, and the responsive mechanism for Cu(I). (B) The responsive mechanism of probe **8**. (C) The structure of probe **9**, the imaging of LO2 cells and BALB/c mice treated with probe **9**. Reproduced with permission from Ref. [44] Copyright 2021, Elsevier B.V.

Since tris[(2‐pyridyl)methyl]amine (TPA) as a Cu receptor was firstly conjugated with a reduced fluorescein by benzyl ether linker to construct a fluorescent probe of Cu(I) by Taki and coworkers in 2010,^[41]^ this has proven to be an efficient strategy to design an activity‐based probe of Cu(I). For example, Chung et al.^[42]^ developed a FRET probe **7** (**FCP‐1**) that consisted of a fluorescein as a donor and a rhodamine fluorophore as an acceptor, which were bridged by TPA and benzyl ether linker (Figure 6A). During the recognition process, upon the formation of the complex of Cu(I) with the tetradentate TPA moiety, it required oxygen and water assistance to promote the oxidative decomposition of benzyl ether bond, thereby generating fluorescent substances. In presence of low concentration of Cu(I), the probe remained intact and exhibited weak emission of fluorescein at 526 nm while strong emission of rhodamine at 576 nm under single‐wavelength excitation at 458 nm, due to the FRET process. High concentration of Cu(I) triggered the oxidative cleavage of the C−O bond of benzyl ether, then FRET from the fluorescein donor to the rhodamine acceptor was inhibited, which decreased the fluorescence emission of rhodamine while enhancing the fluorescence emission of fluorescein. The rapid ratiometric fluorescence change (F_526_/F_576_) exhibited a linear dose response to Cu(I) between 0.01 and 1 μM, independing of the probe concentration, which minimized the interferences from probe concentration and other environmental factors. It also exhibited self‐quenching at high concentration as an inevitable disadvantage of aggregation‐caused quenching fluorophores. In imaging of live cells, it has revealed that oncogene‐driven cell transformation promoted a decrease in the GSH/GSSH ratio (the reduced and oxidized form of glutathione respectively), leading to labile Cu(I) deficiency but unchanged total Cu levels.

TPA was used as a caging unit in an activity‐based Cu(I) probe **8** (**CP1**, Figure 6B).^[43]^ The caged probe **8** remained non‐fluorescent until Cu(I) broke the benzylic ether bond (C−O). The phenolic hydroxyl group of uncaged **8** tended to undergo in situ pinner cyclization to give fluorescent coumarin analogue, leading to 60‐fold fluorescence enhancement. However, this probe responded to both Cu(I) and Cu(II). In addition, probe **8** required a longer time to respond to Cu(I) (Table 1, approximately 90 min), which was not conducive to timely and truthful feedback on Cu metabolism.

Similarly, mitochondria‐targeted NIR probe **9** (**NFL‐TPA**, Figure 6B) developed by Qian et al.^[44]^ in 2021, achieved specific detection of Cu(I). It not only achieved mitochondrial imaging of Cu(I) in living mice, but also evaluated the efficacy of the drug D‐penicillamine, utilized to treat Wilson disease. Probe **9**, using the semi‐heptamethine core as the mitochondrial targeting group, showed a Pearson's colonization coefficient of ~0.81 with rhodamine 123, a commercial mitochondrial fluorophore (Figure 6C). In the bioimaging experiments, NIR fluorescence of probe **9** enhanced by treated with external Cu(I). The emission peak at 732 nm (Table 1) of probe **9**/Cu(I) enabled it to image the distribution of Cu(I) in living mice in vivo and ex vivo.

### 2.2 Copper(II)

Cu exists mainly in the form of Cu(II) outside the cell. Besides, in intracellular certain regions, the Cu species presents as Cu(II), such as, mitochondrial cytochrome *c* oxidase (COX) and superoxide dismutase (SOD).^[27]^ Interestingly, during the process of cuproptosis, the valence states of Cu are sometimes converted mutually in the cell. Moreover, variation of Cu level in biological fluids can be observed in Cu‐relative diseases.^[27]^ Thus, the luminescent Cu(II) probe is a promising tool for the direct measurement of cuproptosis and exchangeable copper pools, which could facilitate the study of Cu metabolism and further offer the possibility of cuproptosis intervention therapy.

#### 2.2.1 Affinity‐based probes for Cu(II)

The advantage of affinity‐based Cu(II) probes is reversibility, but a few probes remain fluorescence turn‐off process.^[45]^ The mechanism of the fluorescence changes can be divided into following three types: (1) disruption of the structural plane of the probe after binding to Cu(II); (2) change of the conformation of the probe after binding to Cu(II) and (3) direct binding to Cu(II) without other changes. The affinity‐based Cu(II) fluorescence turn‐on probes basically belong to the second type.

**Figure 7:**
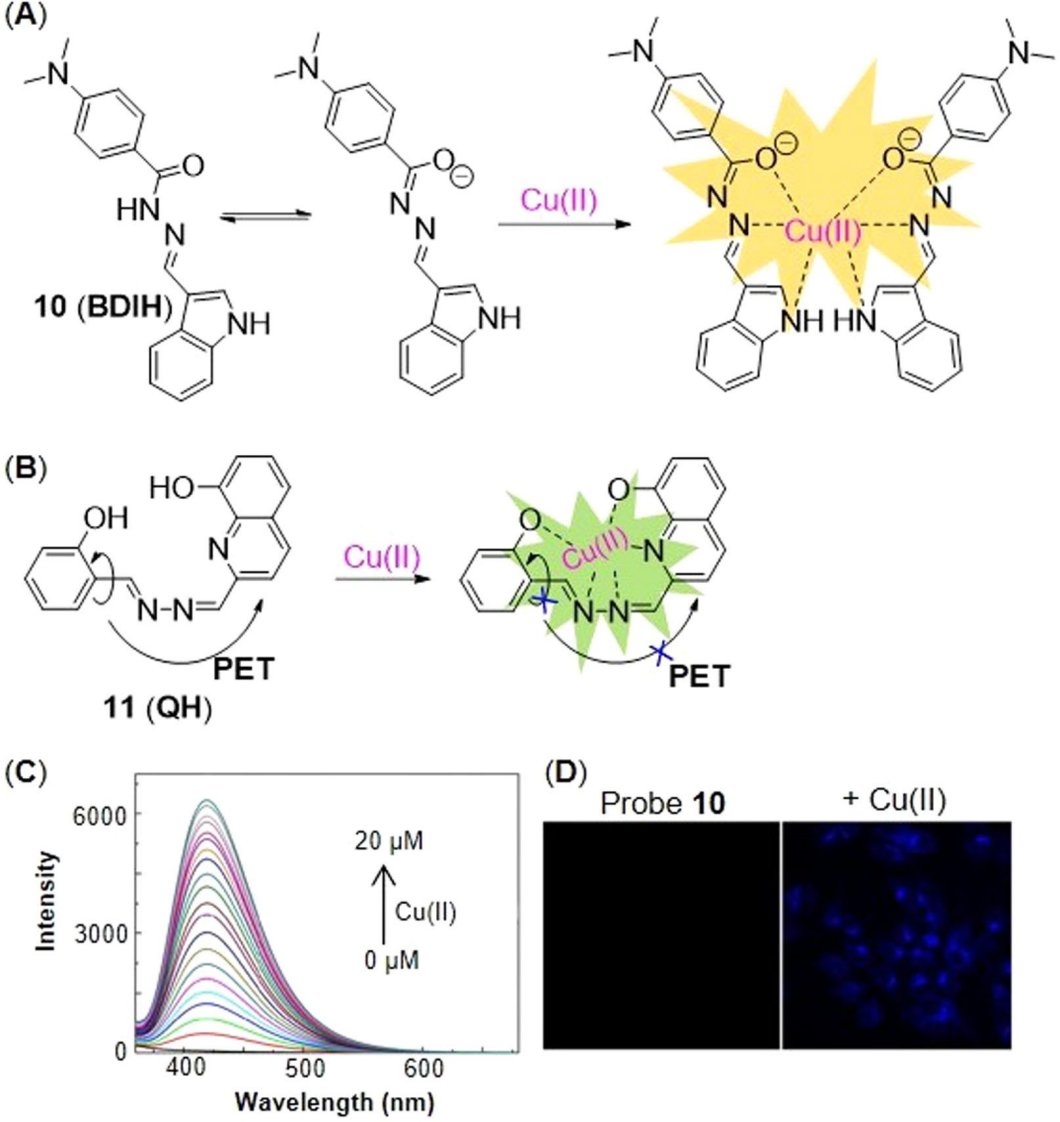
The representing affinity‐based probes for Cu(II). The structures of probe **10** (A), **11** (B), and the responsive mechanisms for Cu(II). Fluorescence spectra (C) and imaging (D) of Hepatoma carcinoma cells treated with probe **10**. Reproduced with permission from Ref. [46] Copyright 2017, Elsevier B.V.

Wu′s group^[46]^ invented an ultrasensitive Cu(II) probe **10** (**BDIH**, Figure 7A) based on a *p*‐dimethylaminobenzamide derivative. It had high stability for at least three months and a detection limit as low as 0.64 nM (Table 2), so far as the lowest detection limit among the fluorescence enhancing Cu probes in this article. The oxygen atom on the carbonyl group, the nitrogen atom on the benzazole and Schiff base effectively chelated Cu(II), which further inhibited the isomerization of −O=C−N to −O−C=N, hence the fluorescence was enhanced at 419 nm. The fluorescence titration spectra showed a remarkable enhancement at 419 nm in response to the increasing concentration of Cu(II) (Figure 7C). The imaging of hepatoma carcinoma cells showed that there was almost no fluorescence in the cells pre‐treated with probe **10**, until addition of Cu(II) (Figure 7D).

**Table 2:** The representative Cu(II) probes.

S. no	Structure of sensors	Luminescence mechanism	λ_ex_/λ_em_ [nm]	Response time	Limit of detection	Cell lines	Refs.
10	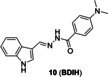	Affinity‐based: /	363/419	8 min	0.64 nM	Hepatoma Carcinoma cells	[46]
11	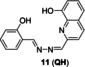	Affinity‐based: intramolecular proton transfer (ESIPT)	352/623	/	8.08 nM	PC‐12 cells	[47]
12	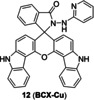	Activity‐based: /	488/520	30 min	88.7 nM	L929 cells	[52]
13	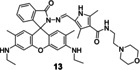	Activity‐based: FRET	360/486	10 min	105 nM	HeLa cells	[53]
14	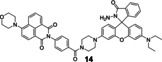	Activity‐based: FRET	420/540	<1 min	18.6 nM	MGC‐803 cells	[54]
15	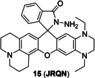	Activity‐based: ICT	580/678	50 s	29 nM	HeLa cells	[55]
16	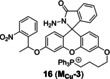	Activity‐based: FRET	455/525	60 min	170 nM	HepG2 cells	[57]
17	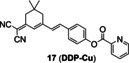	Activity‐based: ICT	520/665	/	36 nM	SH‐SY5Y cells	[60]
18	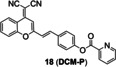	Activity‐based: ICT	560/676	40 min	23 nM	HeLa cells	[61]
19	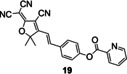	Activity‐based: ICT	580/610	10 min	54 nM	HeLa cells	[62]
20	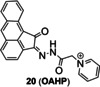	Activity‐based: /	370/490	30 min	18 nM	HepG2 cells	[63]
21	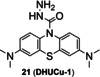	Activity‐based: /	620/686	/	19.1 nM	HeLa cells	[64]
22	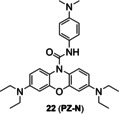	Activity‐based: /	654/669	190 s	1.93 nM	HeLa cells	[24]

A quinoline‐derived Schiff base probe **11** (**QH**, Figure 7B) for Cu(II) was developed by Wang and coworkers,^[47]^ which had a detection limit as low as 8.08 nM (Table 2) and a binding ratio of 1 : 1. In addition, the probe had a colorimetric selectivity that was visible to the naked eye. The addition of Cu(II) could change the solution from colorless to yellow‐green. Probe **11** was essentially nonfluorescent on account of cis‐trans isomerization around C=N bond and the PET process that was from the nitrogen near phenol to naphthyl fluorophore. The chelation of Cu(II) with probe **11** inhibited the PET process and the isomerization of C=N bond, which resulted in fluorescence signal increasing. Due to the presence of the photoinduced proton transfer effect, the enol type of probe **11** emitted at 352 nm, while the ketone interconversion isomer (hydrogen atom transferred from the phenol hydroxyl group to the nitrogen atom of the Schiff base) emitted at 623 nm.

#### 2.2.2 Activity‐based probes for Cu(II)

Fluorescence turn‐on Cu(II) probes are usually activity‐based mechanism which conduct uncaging reactions or other reactions to induce fluorescence changes. Activity‐based probes effectively exclude false background signals due to irreversible chemical reactions.^[48]^

**Figure 8:**
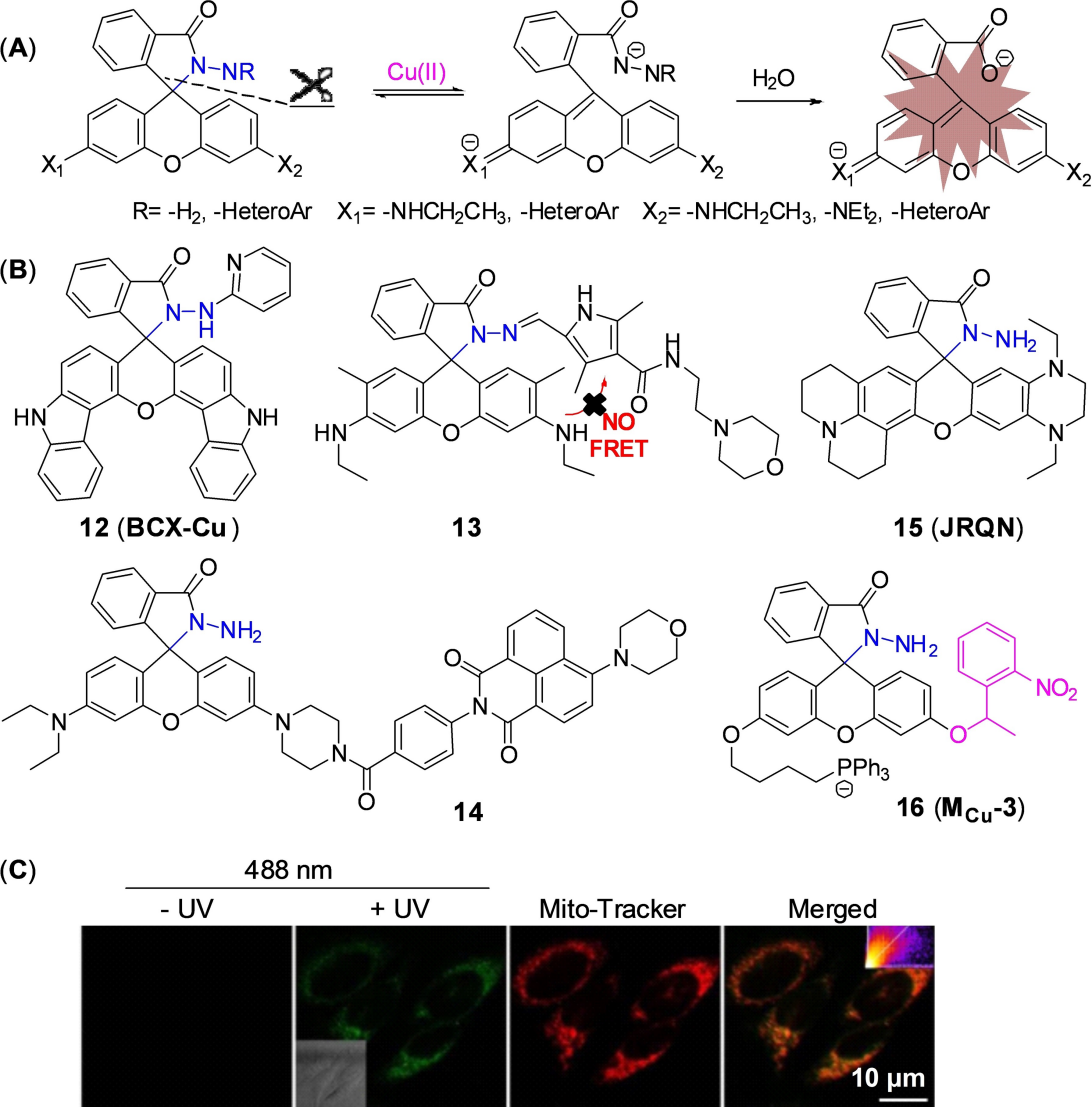
The Cu(II) probes with rhodamine spirolactam as reaction site. (A) Mechanism for detection of Cu(II) by rhodamine based probes: Cu(II)‐initiated ring‐opening reaction of rhodamine spirolactam. (B) The structures of probes **12**~**16**. (C) Bioimaging results of HepG2 stained with probe **16**. Reproduced with permission from Ref. [57] Copyright 2017, Elsevier B.V.

Cu(II) can initiate ring‐opening of rhodamine spirolactam to generate fluorescence. Due to excellent photophysical properties, such as high quantum yield and stability, rhodamine and its derivatives are often used to design chemical fluorescent probes. In the 1990s, Dujols and coworkers^[49]^ designed the first chemical probe based on rhodamine B for Cu(II). Rhodamine B hydrazide in ring‐closing form remains extremely weak fluorescence. Cu(II) induces a specific ring‐opening reaction of spirolactam followed by a cascade hydrolysis reaction, which restores strong fluorescence of rhodamine B (Figure 8A).^[50]^ Based on this mechanism, an increasing number of Cu(II) probes derived from rhodamine have emerged.^[51]^ For example, Huang et al.^[52]^ reported a fluorescent probe **12** (**BCX‐Cu**, Figure 8B) based on the scaffold of biscarbazole‐fused xanthene hybrid, which had high selectivity and sensitivity to Cu(II). In addition, it had a detection limit of 88.7 nM (Table 2) and a dissociation constant of (19.233±0.385)×10^−6^ M. In the absence of Cu(II), probe **12** had absorption bands at 319 nm and 334 nm, which were characteristic of the carbazole moiety. When Cu(II) was added, there was an absorption band at 536 nm and an emission peak at 570 nm.

As previously reported, FRET process allows a fluorescent probe to detect Cu in a ratiometric manner. Wu′s group^[53]^ developed a lysosome‐targeted ratiometric fluorescent probe **13** (Figure 8B) consisting of a pyrrole moiety as a FRET donor and a rhodamine moiety as a FRET acceptor. In absence of Cu(II), the free probe emitted fluorescence at 486 nm, while treated with Cu(II), the FRET process was activated by ring‐opening of spirolactam and deprotonation of pyrrole followed by coordination, which emitted the characteristic fluorescence of rhodamine at 552 nm. It responded rapidly (Table 2, <15 min) to Cu(II) at pH (4.0~8.0). Besides, the morpholine handle was introduced for lysosome‐targeting. Tang and coworkers^[54]^ developed a fast responsive (Table 2, <1 min) ratiometric fluorescent probe **14** consisting of rhodamine as a FRET donor and naphthalimide derivative as a FRET acceptor (Figure 8B). Rapid Cu(II) response of probe **14** was beneficial for timely monitoring the process of cuproptosis. The naphthalimide derivative had a broad absorption spectrum (450–650 nm), which partially overlapped with that of rhodamine (500–560 nm), so the combination of them was beneficial for the construction of FRET based probes.

Since mitochondrion is often described as ‘the power plants of life’ and strongly associated with cuproptosis, mitochondrion‐targeting is very important in the investigation of cuproptosis. Gong et al.^[55]^ utilized the cationic xanthene as a mitochondrion‐targeting group as negative potential in the mitochondrial membrane,^[56]^ introduced the electron‐giving group 1,4‐diethylpiperazine to the rhodamine backbone, and further designed a probe **15** (**JRQN**, Figure 8B). It had large stokes shift as well as NIR emission wavelength (Table 2, 678 nm) and excellent properties, such as high photostability, high selectivity, fast response (50 s) as well as high sensitivity. However, the probe could react with Cu(II) in the cytoplasm before reaching the mitochondria, resulting in a false mitochondrial Cu(II) signal. A caged probe **16** (**M_cu_‐3**, Figure 8B) that was designed by our group^[57]^ tackled this issue well. Apart from rhodamine dye, it also was equipped with a famous mitochondrial targeting moiety (triphenylphosphonium salt, TPP^+^) and a photocaged group (2‐nitrobenzyloxy unit). The modifications were not only essential for accurate imaging of mitochondrial Cu(II), but also increased the cellular permeability of **16**. The 2‐nitrobenzyloxy unit prevented cytoplasmic Cu(II) from hydrolyzing hydrazide, ensuring non‐fluorescent **16** targeting to mitochondria. When **16** entered the mitochondria, UV irradiation uncaged the photo‐labile group and then mitochondrial Cu(II) activated **16** to subsequently emit fluorescence, providing spatial and temporal visualization of mitochondrial Cu(II) imaging (Figure 8C). Thus, probe **16** contributed to accurately revealing the mechanism of cuproptosis due to the design strategy.

**Figure 9:**
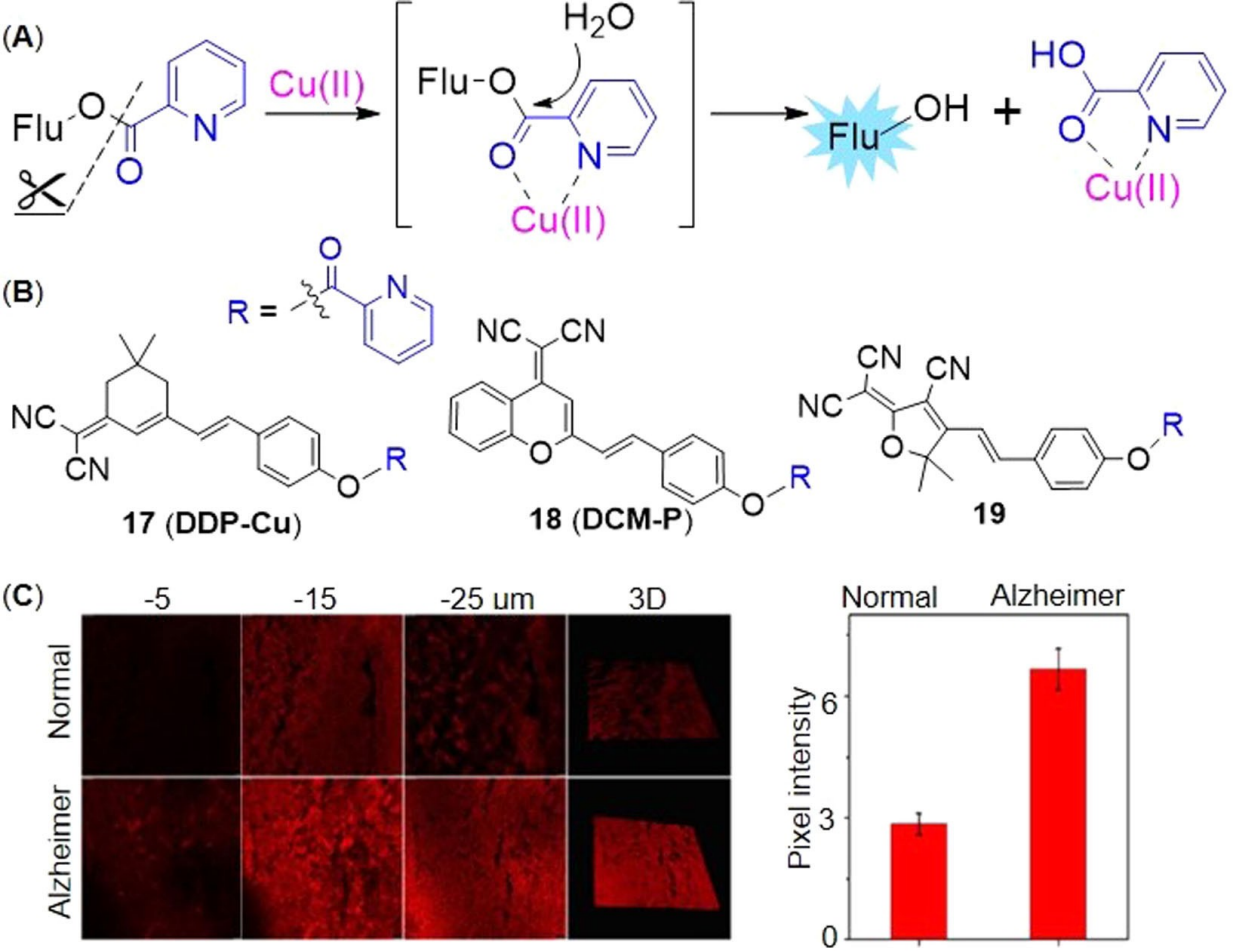
The Cu(II) probes with picolinate as the caged group. (A) Typical mechanism for the detection of Cu(II): Cu(II)‐initiated hydrolysis reaction (B) The structures of probes **17**–**19** (C) Bioimaging results of mouse brain tissue slices stained with probe **17**. Reproduced with permission from Ref. [60] Copyright 2022, Elsevier B.V.

Cu(II) can initiate hydrolysis of picolinate. Since 2‐pyridine carbonyl was firstly adopted as a Cu(II)‐responsive caging group to design a fluorescence turn‐on Cu(II) probe^[58]^ by Zhou et al. in 2011, it has become a typical strategy for Cu(II) sensing. 2‐Pyridine carbonyl has a strong electron‐withdrawing effect, which can effectively quench the fluorescence. Cu(II) catalyzes the specific hydrolysis of picolinate to restore the strong fluorescence of 7‐hydroxycoumarin (Figure 9A). Because the hydrolytic product picolinic acid had a higher affinity than the probe and competed with the probe to form a complex with Cu(II),^[59]^ it is a stoichiometric reaction.

Similarly, the strategy was applied to fluorescent probes **17**–**19** by caging fluorophore dicyanoisophorone derivative, dicyanomethylene‐4*H*‐pyran derivative and tricyanofuran respectively. Li′s group^[60]^ constructed the fluorescent probe **17** (**DDP‐Cu**) with a NIR emission peak at 665 nm (Table 2) as well as a detection limit of 36 nM (Figure 9B). It had good photostability by continuous light exposure for 1.5 h, low cytotoxicity and deep tissue‐penetrating ability. During imaging Cu(II) in the brain tissue slices of Alzheimer's disease model mice, it showed a three‐fold enhancement in fluorescence intensity, compared with normal ones (Figure 9C). Probe **18** (**DCM‐P**) developed by Gu and coworkers^[61]^ had a large stokes shift (116 nm) and good photostability. When Cu(II) was added, the probe solution altered from yellow to purple as seen by the naked eyes. In addition, the probe **19** ^[62]^ also showed high selectivity and sensitivity (Table 2, detection limit: 54 nM) to Cu(II), which could be used to visualize Cu(II) in living cells. The addition of Cu(II) significantly enhanced the fluorescence at 610 nm, and the reaction was completed within 10 min.

**Figure 10:**
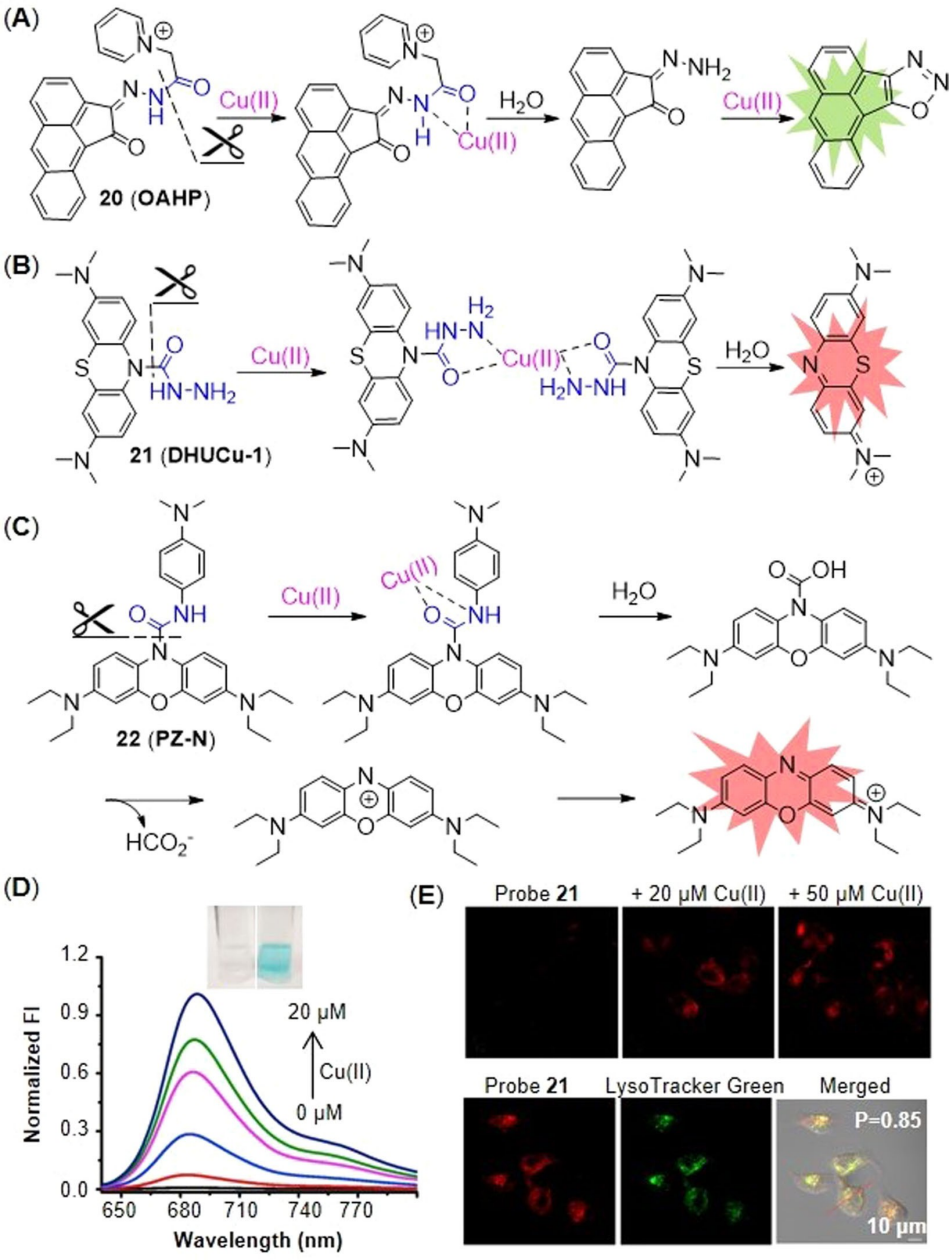
The probes with C−N bond as the reaction site with Cu(II). (A) The structure of probe **20** and the mechanism for detection of Cu(II). (B) The structure of probe **21** and the mechanism for detection of Cu(II). (C) The structure of probe **22** and the mechanism for detection of Cu(II). (D) Fluorescence spectra of probe **21** with various concentrations of Cu(II) and the color change before (left)/after (right) addition of Cu(II). (E) Fluorescent imaging of HeLa cell treated with probe **21** in presence of Cu(II). Reproduced with permission from Ref. [64] Copyright 2022, Elsevier B.V.

Cu(II) can initiate the hydrolysis of C−N bond and cascade reactions. By forming a Cu complex, Cu(II) activates C=O bond and facilitates the specific hydrolysis of picolinate, which obviously has the potential to hydrolyze other groups, such as hydrazide and urea, and further uncage the fluorophore. Okamoto et al.^[63]^ applied this strategy to design a Cu(II) probe **20** (**OAHP**) based on aceanthrene. The fluorescence of aceanthrene was quenched by the hydrazide moiety isomerization. The amide group of probe **20** was hydrolyzed by the aid of Cu(II) to give aceanthraquinone monohydrazone. The hydrazone moiety of intermediate was readily oxidized by Cu(II) to further form diazo compound which subsequently underwent intramolecular cyclization to give a strongly fluorescent aceathrene‐oxadiazole derivatives (Figure 10A).

The strategy was also applied to design a lysosome‐targeting NIR probe **21** (**DHUCu‐1**) of Cu(II) based on methylene blue (MB, λ_em_=680 nm) by Chen and coworkers^[64]^ (Figure 10B). Compared with previously reported probes with lysosome targeting group,[^65^, ^66^] probe **21** has improved water solubility and required merely 1 % alcohol as the co‐solvent. During the process of detecting Cu(II), the recognition group formyl hydrazide and Cu(II) formed the coordination compound, which was unstable in water and easily hydrolyzed to leucomethylene blue and then MB. The addition of Cu(II) not only significantly enhanced the fluorescence intensity, but also enabled us to observe the color change with the naked eye (Figure 10D). In cell imaging, probe **21** showed good response to Cu(II) in lysosomes (Figure 10E).

Regrettably, the probe **21** with MB as the backbone did not show fast response or high sensitivity to Cu(II). Li′s group^[24]^ developed another NIR fluorescent probe **22** (**PZ‐N**) that based on fluorophore phenoxiazine with similarly photophysical properties of MB. It exhibited a fast response of 40 s (Table 2) and a low detection limit of 1.93 nM through two‐step reaction. At first, Cu(II) bound to the amide moiety of the probe and then formed a highly unstable complex. Then, hydrolysis of carbonyl group led to the cleavage of C−O bond and further produced unstable carbamic acid. Finally, carbamic acid released HCO_2_^−^ and generated an unstable intermediate that rapidly formed the strongly fluorescent phenoxiazine (Figure 10C). The probe showed good selectivity and stability in presence of various interference factors, such as glutathione, aldehyde, wide range of pH (2–10). Therefore, probe **22** remained the potential compound to investigate cuproptosis.

To sum up, in the past decade, there are two types of fluorescent probes toward detecting Cu, such as affinity‐based and activity‐based probes, which are equipped with the different recognition groups. It is worth noting that these recognition groups contain heteroatoms such as oxygen, nitrogen, or sulfur atoms. Affinity‐based probes rely on the hetero‐atom containing recognition groups with high affinity to Cu, such as thioether, peptide residues, phosphonium sulfides as well as TPA. Activity‐based probes rely on the recognition groups with high affinity and specific activity to Cu, such as amide and picolinate. Subsequently, the specific interactions between the recognition groups and analytes (Cu ions) induce the changes in optical properties, such as fluorescent wavelength, fluorescent intensity, and fluorescence lifetime. Reversible detection of Cu is an advantage of the affinity‐based probes, which means that it can respond to the fluctuations of labile copper pools in a period. Although activity‐based probes generally have higher selectivity as well as sensitivity, it may not be able to detect temporal alteration of labile Cu well because of the irreversible process and even slow response. They can detect Cu in organelles such as *Golgi* apparatus, endoplasmic reticulum, mitochondria, and lysosomes, but there are still few probes that can simultaneously detect Cu in different regions.

## 3 Summary and Outlook

In 2022, Tsvetkov and colleagues firstly proposed a new programmed cell death, cuproptosis, which is caused by excessive Cu and closely related to mitochondria. Different from the programmed cell death reported previously, such as apoptosis and ferroptosis, cuproptosis may provide a new perspective for clinical treatment of diseases. However, the specific mechanism and morphological characteristics of cuproptosis still remains unclear. Therefore, a convenient tool to further explore the process is necessary.

In recent years, small‐molecule fluorescent probes of Cu have experienced an explosive development due to their advantages in low cost, non‐invasiveness, high sensitivity, and accuracy. Nevertheless, most of them remain fluorescence turn‐off process due to the paramagnetic effect of Cu, which cannot accurately study the physiological process of Cu because fluorescence quenching may be caused by various environmental factors within the cell. It is worth noting that we have summarized the advantages and disadvantages of fluorescence turn‐on probes of Cu in the past decade, and found that they were not yet well used for the study of cuproptosis. Therefore, based on the known process of cuproptosis, we proposed some perceptions on the new generation of Cu probes from the design to application.

Firstly, a fluorophore largely determines the photophysical properties of the probes. Neonatal probes should choose a fluorophore with following properties: high fluorescence quantum yield, high stability, a large stokes shift as well as a balance between hydrophilicity and lipophilicity to achieve the better cell penetration and intracellular water‐solubility simultaneously. In addition, NIR fluorophores can overcome self‐fluorescence and improve higher resolution, making them a good tool for imaging in vivo. Besides ratiometric fluorescent probes and FLIM, there is an urgent need for new techniques to overcome concentration‐dependence as the inherent disadvantage of traditional fluorescence turn‐on probes. Developing new recognition groups is also important for perusing higher selectivity, sensitivity, and shorter response time. Secondly, it should achieve specific detection of various oxidative state of Cu and even simultaneous detection, which is not achievable by the known probes. This function can help us better understand the metabolic pathways and spatiotemporal distributions of endogenous Cu species. Thirdly, it should be able to achieve organelle‐targeting detection and even simultaneous detection of Cu fluctuations between different organelles, because these organelles have a clear division of labor in Cu metabolism. Lysosome discharges excessive Cu into bile. The *Golgi* apparatus is a key organelle for Cu homeostasis. Mitochondrial respiration can regulate Cu‐induced cell death. Fine‐Tuning of its lipophilicity and super resolution microscopy to morphologically identify the organelles are possible solutions. Fourthly, to understand Cu signaling pathways clearly and comprehensively in cuproptosis, multifunctional fluorescent probes need to detect multiple analytes simultaneously and identify cuproptosis‐related protein/enzymes. For example, activity‐based protein profiling (ABPP) has the potential to identify Cu‐triggered labeling of targets of interest. In addition, new strategy inspired by the research of cuproptosis may disclose pathological research on other Cu disorders. Finally, in terms of its future applications, the proposal of cuproptosis may provide some new perspectives for the treatment of diseases caused by Cu disorder. Given thoroughly understand the disease mechanisms and cuproptosis, intervention of cuproptosis may become a new disease treatment method. For example, we may use cuproptosis to promote cancer cell death and block cell death accurately, effectively, and quickly by inhibiting upstream factors that regulate cuproptosis in neurodegenerative diseases. In addition, the combination of cuproptosis and other programmed cell death, such as ferroptosis, may also be a potential method of disease treatment.

Futhermore, we hope that the specific mechanism of cuproptosis can be clearly revealed and intervention of cuproptosis can also be applied to related disease's therapy in the near future.

## Conflict of interest

The authors declare no conflict of interest.

4

## Biographical Information

*Ting‐en Peng received her Bachelor of Science degree in chemistry from the Jiaying University in 2021 under the supervision of Professor Guiting Chen. She has been studying for a Master's degree in Organic Chemistry since 2021 at the Institute of Advanced Materials, Nanjing Tech University under the supervision of Professor Lin Li. Her current research interest are small‐molecule fluorogenic probes*.

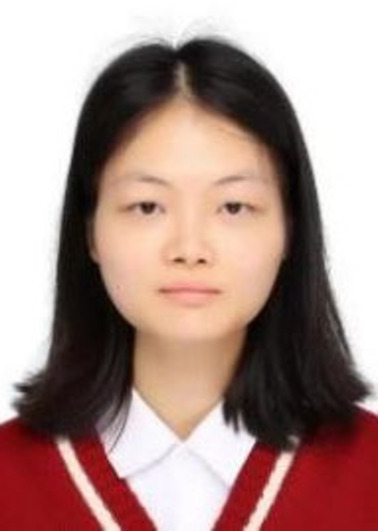

## Biographical Information

*Feng Qiu received his BS from Qilu University of Technology in 2021. He is currently a graduate student in Nanjing Tech University under the supervision of Assoc. Professor Xiamin Cheng. His research interest focuses on developing fluorescent probes for live cell imaging*.

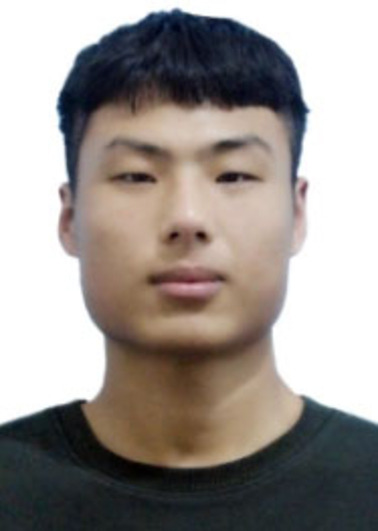

## Biographical Information

*Yunwei Qu received her BS and MS from Nanjing Tech University in 2019 and 2022, respectively. Currently, she is working as a PhD student at Xiamen University with Professor Lin Li. Her research interest mainly focuses on the synthesis and biological application of small‐molecule fluorogenic probes*.

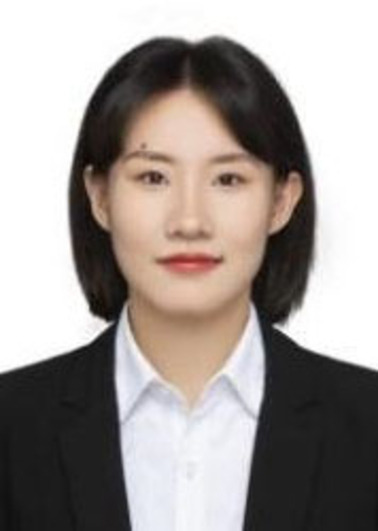

## Biographical Information

*Changmin Yu received his PhD from South China University of Technology in 2013. Afterwards, he started postdoctoral studies with Professor Shao Q. Yao at the National University of Singapore (NUS) from 2014. He got his professor position in Nanjing Tech University in 2018. His research interest focuses on organic and biomedical photonics for biological applications including imaging and protein delivery*.

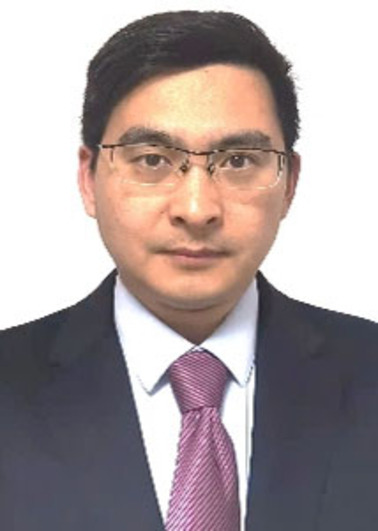

## Biographical Information

*Xiamin Cheng received his PhD from National University of Singapore (NUS) under Prof. Shao Q. Yao's supervision in 2014. Then he got postdoctoral training in Prof. Bin Liu's group at NUS. After that, he obtained his current position as associate professor at Nanjing Tech University in 2017. His research interest focuses on fluorescent materials for biological applications including imaging and drug delivery*.

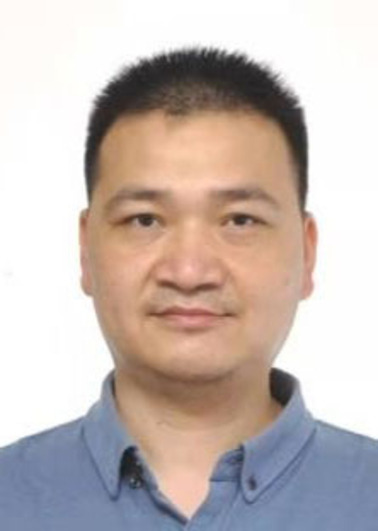

## Biographical Information

*Lin Li received his BSc and PhD in Chemistry from Anhui University in 2004 and 2009, respectively. He carried out postdoctoral studies with Professor Shao Q. Yao at NUS. He got his professor position at Nanjing Tech University in 2015, and adjunct professor position in Northwestern Polytechnical University (NPU) in 2018. Then, he moved to Xiamen University (XMU) in 2023 as a professor. His research group focuses on mitochondrial information and health engineering*.

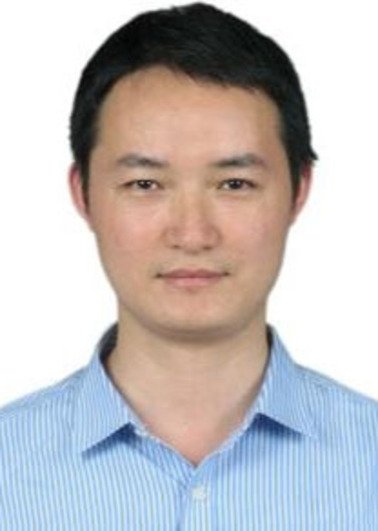

## Data Availability

Data sharing is not applicable to this article as no new data were created or analyzed in this study.

## References

[1] T. Hirayama , A. Miki , H. Nagasawa , Metallomics 2019, 11, 111–117. 30215439 10.1039/c8mt00212f

[2] S. J. Dixon , K. M. Lemberg , M. R. Lamprecht , R. Skouta , E. M. Zaitsev , C. E. Gleason , D. N. Patel , A. J. Bauer , A. M. Cantley , W. S. Yang , B. Morrison III , B. R. Stockwell , Cell 2012, 149, 1060–1072. 22632970 10.1016/j.cell.2012.03.042 PMC3367386

[3] E. J. Ge , A. I. Bush , A. Casini , P. A. Cobine , J. R. Cross , G. M. DeNicola , Q. P. Dou , K. J. Franz , V. M. Gohil , S. Gupta , S. G. Kaler , S. Lutsenko , V. Mittal , M. J. Petris , R. Polishchuk , M. Ralle , M. L. Schilsky , N. K. Tonks , L. T. Vahdat , L. Van Aelst , D. Xi , P. Yuan , D. C. Brady , C. J. Chang , Nat. Rev. Cancer 2022, 22, 102–113. 34764459 10.1038/s41568-021-00417-2 PMC8810673

[4] T. Nevitt , H. Ohrvik , D. J. Thiele , Biochim. Biophys. Acta 2012, 1823, 1580–1593. 22387373 10.1016/j.bbamcr.2012.02.011 PMC3392525

[5] Y. Wang , V. Hodgkinson , S. Zhu , G. A. Weisman , M. J. Petris , Adv. Nutr. 2011, 2, 129–137. 22332042 10.3945/an.110.000273 PMC3065767

[6] T. Tsang , C. I. Davis , D. C. Brady , Curr. Biol. 2021, 31, R421–R427. 33974864 10.1016/j.cub.2021.03.054

[7] Y. Duan , H. Tang , Y. Guo , Z. Song , M. Peng , Y. Yan , Chin. Chem. Lett. 2014, 25, 1082–1086.

[8] N. M. Garza , A. B. Swaminathan , K. P. Maremanda , M. Zulkifli , V. M. Gohil , Trends Endocrinol. Metab. 2023, 34, 21–33. 36435678 10.1016/j.tem.2022.11.001 PMC9780195

[9] M. Moriya , Y. H. Ho , A. Grana , L. Nguyen , A. Alvarez , R. Jamil , M. L. Ackland , A. Michalczyk , P. Hamer , D. Ramos , S. Kim , J. F. Mercer , M. C. Linder , Am. J. Physiol. 2008, 295, C708-C721. 10.1152/ajpcell.00029.2008 PMC2544443 18579803

[10] J. A. Cotruvo Jr. , A. T. Aron , K. M. Ramos-Torres , C. J. Chang , Chem. Soc. Rev. 2015, 44, 4400–4414. 25692243 10.1039/c4cs00346b PMC4478099

[11] C. J. Fahrni , Curr. Opin. Chem. Biol. 2013, 17, 656–662. 23769869 10.1016/j.cbpa.2013.05.019 PMC3774042

[12] J. Calvo , H. Jung , G. Meloni , IUBMB Life 2017, 69, 236–245. 28296007 10.1002/iub.1618

[13] J. R. Prohaska , Am. J. Clin. Nutr. 2008, 88, 826S–829S. 18779302 10.1093/ajcn/88.3.826S PMC2799992

[14] S. La Fontaine , J. F. Mercer , Arch. Biochem. Biophys. 2007, 463, 149–167. 17531189 10.1016/j.abb.2007.04.021

[15] A. T. Aron , K. M. Ramos-Torres , J. A. Cotruvo Jr. , C. J. Chang , Acc. Chem. Res. 2015, 48, 2434–2442. 26215055 10.1021/acs.accounts.5b00221 PMC4542203

[16] G. G. Chan , C. M. Koch , L. H. Connors , J. Proteome Res. 2017, 16, 1659–1668. 28196416 10.1021/acs.jproteome.6b00998

[17] M. Valko , H. Morris , T. D. M. Cronin , Curr. Med. Chem. 2005, 12, 1161–1208. 15892631 10.2174/0929867053764635

[18] P. Tsvetkov , S. Coy , B. Petrova , M. Dreishpoon , A. Verma , M. Abdusamad , J. Rossen , L. Joesch-Cohen , R. Humeidi , R. D. Spangler , J. K. Eaton , E. Frenkel , M. Kocak , S. M. Corsello , S. Lutsenko , N. Kanarek , S. Santagata , T. R. Golub , Science 2022, 375, 1254–1261. 35298263 10.1126/science.abf0529 PMC9273333

[19] Y. Wang , L. Zhang , F. Zhou , Cell. Mol. Immunol. 2022, 19, 867–868. 35459854 10.1038/s41423-022-00866-1 PMC9338229

[20] C. E. Outten , T. V. O′Halloran , Science 2001, 292, 2488–2492. 11397910 10.1126/science.1060331

[21] Q. Fu , J. Ye , J. Wang , N. Liao , H. Feng , L. Su , X. Ge , H. Yang , J. Song , Small 2021, 17, 2008061. 10.1002/smll.202008061 34081397

[22] S. Schmollinger , S. Chen , D. Strenkert , C. Hui , M. Ralle , S. S. Merchant , Proc. Natl. Acad. Sci. USA 2021, 118, e2026811118. 33879572 10.1073/pnas.2026811118 PMC8072209

[23] Z. Li , J. Hou , S. Wang , L. Zhu , X. He , J. Shen , Coord. Chem. Rev. 2022, 469, 214695.

[24] Y. Shen , W. Zheng , Y. Yao , D. Wang , G. Lv , C. Li , Chem. Asian J. 2020, 15, 2864–2867. 32720435 10.1002/asia.202000783

[25] K. H. Jung , E. T. Oh , H. J. Park , K. H. Lee , Biosens. Bioelectron. 2016, 85, 437–444. 27208475 10.1016/j.bios.2016.04.101

[26] H. Xu , S. Yao , Y. Chen , C. Zhang , S. Zhang , H. Yuan , Z. Chen , Y. Bai , T. Yang , Z. Guo , W. He , Inorg. Chem. 2021, 60, 18567–18574. 34826221 10.1021/acs.inorgchem.1c01779

[27] E. Falcone , M. Okafor , N. Vitale , L. Raibaut , A. Sour , P. Faller , Coord. Chem. Rev. 2021, 433, 213727.

[28] X. Q. Yi , Y. F. He , Y. S. Cao , W. X. Shen , Y. Y. Lv , ACS Sens. 2019, 4, 856–864. 30868875 10.1021/acssensors.8b01240

[29] K. Rurack , U. Resch-Genger , Chem. Soc. Rev. 2002, 31, 116–127. 12109205 10.1039/b100604p

[30] Y. Wang , H. Yu , Y. Zhang , C. Jia , M. Ji , Dyes Pigm. 2021, 190, 109284.

[31] M. L. Giuffrida , E. Rizzarelli , G. A. Tomaselli , C. Satriano , G. Trusso Sfrazzetto , Chem. Commun. 2014, 50, 9835–9838. 10.1039/c4cc02147a 24827742

[32] C. Satriano , G. T. Sfrazzetto , M. E. Amato , F. P. Ballistreri , A. Copani , M. L. Giuffrida , G. Grasso , A. Pappalardo , E. Rizzarelli , G. A. Tomaselli , R. M. Toscano , Chem. Commun. 2013, 49, 5565–5567. 10.1039/c3cc42069h 23673488

[33] X. Cao , W. Lin , W. Wan , Chem. Commun. 2012, 48, 6247–6249. 10.1039/c2cc32114a 22595897

[34] M. T. Morgan , D. Bourassa , S. Harankhedkar , A. M. McCallum , S. A. Zlatic , J. S. Calvo , G. Meloni , V. Faundez , C. J. Fahrni , Proc. Natl. Acad. Sci. USA 2019, 116, 12167–12172. 31160463 10.1073/pnas.1900172116 PMC6589653

[35] Y. Bai , D. Liu , Z. Han , Y. Chen , Z. Chen , Y. Jiao , W. He , Z. Guo , Sci. China Chem. 2018, 61, 1413–1422.

[36] J. T. Hou , B. Wang , Y. Zhang , B. Cui , X. Cao , M. Zhang , Y. Ye , S. Wang , Chem. Commun. 2020, 56, 2759–2762. 10.1039/c9cc09652c 32022003

[37] W. Zhang , Z. Ma , L. Du , M. Li , Analyst 2014, 139, 2641–2649. 24755654 10.1039/c3an02379f

[38] M. Priessner , P. A. Summers , B. W. Lewis , M. Sastre , L. Ying , M. K. Kuimova , R. Vilar , Angew. Chem. Int. Ed. 2021, 60, 23148–23153. 10.1002/anie.202109349 PMC8596571 34379368

[39] X. Liu , B. Yu , Y. Shen , H. Cong , Coord. Chem. Rev. 2022, 468, 214609.

[40] X. Chai , W. Zhu , Q. Meng , T. Wang , Chin. Chem. Lett. 2021, 32, 210–213.

[41] M. Taki , S. Iyoshi , A. Ojida , I. Hamachi , Y. Yamamoto , J. Am. Chem. Soc. 2010, 132, 5938–5939. 20377254 10.1021/ja100714p

[42] C. Y. Chung , J. M. Posimo , S. Lee , T. Tsang , J. M. Davis , D. C. Brady , C. J. Chang , Proc. Natl. Acad. Sci. USA 2019, 116, 18285–18294. 31451653 10.1073/pnas.1904610116 PMC6744846

[43] Z. Hu , J. Hu , H. Wang , Q. Zhang , M. Zhao , C. Brommesson , Y. Tian , H. Gao , X. Zhang , K. Uvdal , Anal. Chim. Acta 2016, 933, 189–195. 27497012 10.1016/j.aca.2016.05.031

[44] X. Qian , W. Zhu , H. Yu , Y. Xu , W. Liu , H. Wang , Y. Liu , Dyes Pigm. 2021, 194, 109561.

[45] G. Sivaraman , M. Iniya , T. Anand , N. G. Kotla , O. Sunnapu , S. Singaravadivel , A. Gulyani , D. Chellappa , Coord. Chem. Rev. 2018, 357, 50–104.

[46] P. Huang , H. Fang , J. Xiong , F. Wu , Spectrochim. Acta Part A 2017, 173, 264–269. 10.1016/j.saa.2016.09.011 27673495

[47] P. Wang , J. Fu , K. Yao , Y. Chang , K. Xu , Y. Xu , Sens. Actuators B 2018, 273, 1070–1076.

[48] J. Chan , S. C. Dodani , C. J. Chang , Nat. Chem. 2012, 4, 973–984. 23174976 10.1038/nchem.1500 PMC4096518

[49] Y. Xiang , A. Tong , Org. Lett. 2006, 8, 1549–1552. 16597107 10.1021/ol060001h

[50] M. K. Lee , P. Rai , J. Williams , R. J. Twieg , W. E. Moerner , J. Am. Chem. Soc. 2014, 136, 14003–14006. 25222297 10.1021/ja508028h PMC4195381

[51] M. Dong , T. Ma , A. Zhang , Y. Dong , Y. Wang , Y. Peng , Dyes Pigm. 2010, 87, 164–172.

[52] K. Huang , D. Han , X. Li , M. Peng , Q. Qiu , D. Qin , J. Fluoresc. 2019, 29, 727–735. 31104269 10.1007/s10895-019-02393-1

[53] W. Wu , H. Wu , R. Zhong , Y. Wang , Z. Xu , X. Zhao , Z. Xu , Y. Fan , Spectrochim. Acta Part A 2019, 212, 121–127. 10.1016/j.saa.2018.12.041 30616165

[54] J. Tang , S. Ma , D. Zhang , Y. Liu , Y. Zhao , Y. Ye , Sens. Actuators B 2016, 236, 109–115.

[55] J. Gong , C. Liu , X. Jiao , S. He , L. Zhao , X. Zeng , RSC Adv. 2020, 10, 38038–38044. 35515182 10.1039/d0ra05835a PMC9057182

[56] B. Kadenbach , Biochim. Biophys. Acta 2003, 1604, 77–94. 12765765 10.1016/s0005-2728(03)00027-6

[57] L. Wang , B. Chen , P. Peng , W. Hu , Z. Liu , X. Pei , W. Zhao , C. Zhang , L. Li , W. Huang , Chin. Chem. Lett. 2017, 28, 1965–1968.

[58] Z. Zhou , N. Li , A. Tong , Anal. Chim. Acta 2011, 702, 81–86. 21819863 10.1016/j.aca.2011.06.041

[59] C. Zhao , P. Feng , J. Cao , X. Wang , Y. Yang , Y. Zhang , J. Zhang , Y. Zhang , Org. Biomol. Chem. 2012, 10, 3104–3109. 22402504 10.1039/c2ob06980f

[60] Z. Zhou , S. Chen , Y. Huang , B. Gu , J. Li , C. Wu , P. Yin , Y. Zhang , H. Li , Biosens. Bioelectron. 2022, 198, 113858. 34871835 10.1016/j.bios.2021.113858

[61] B. Gu , L. Huang , Z. Xu , Z. Tan , H. Meng , Z. Yang , Y. Chen , C. Peng , W. Xiao , D. Yu , H. Li , Sens. Actuators B 2018, 273, 118–125.

[62] K. H. Nguyen , Y. Hao , K. Zeng , X. Wei , S. Yuan , F. Li , S. Fan , M. Xu , Y. Liu , J. Photochem. Photobiol. A 2018, 358, 201–206.

[63] Y. Okamoto , N. Kishikawa , M. Hagimori , M. El-Maghrabey , S. Kawakami , N. Kuroda , Anal. Chim. Acta 2022, 1217, 340024. 35690425 10.1016/j.aca.2022.340024

[64] Y. Chen , Z. Long , C. Wang , J. Zhu , S. Wang , Y. Liu , P. Wei , T. Yi , Dyes Pigm. 2022, 204, 110472.

[65] M. L. Giuffrida , G. Trusso Sfrazzetto , C. Satriano , S. Zimbone , G. A. Tomaselli , A. Copani , E. Rizzarelli , Inorg. Chem. 2018, 57, 2365–2368. 29431435 10.1021/acs.inorgchem.7b02720

[66] N. Ahmed , W. Zareen , D. Zhang , X. Yang , Y. Ye , Spectrochim. Acta Part A 2022, 264, 120313. 10.1016/j.saa.2021.120313 34474223

